# Trigeminal neuralgia

**DOI:** 10.1186/1129-2377-16-S1-A42

**Published:** 2015-09-28

**Authors:** Andrea Truini

**Affiliations:** Department of Neurology and Psychiatry, University Sapienza, Rome, Italy

The International Association for the Study of Pain (IASP) defines trigeminal neuralgia as a sudden, unilateral, severe, brief, stabbing, recurrent episode of pain in the distribution of one or more branches of the trigeminal nerve. Trigeminal neuralgia (TN) can be distinguished in classical TN and symptomatic TN[1]. Classical TN is due to a vascular compression of the trigeminal root by tortuous or aberrant vessels. Symptomatic TN can be related to cerebello-pontine angle tumours which compress the trigeminal nerve root. Multiple sclerosis (MS) is typically associated with TN (2-4% of patients with TN)[2].

Whether produced by multiple sclerosis or chronic compression exerted by a blood vessel or a benign tumour, demyelination increases the susceptibility of the nerve fibres to ectopic excitation, ephaptic transmission, and high-frequency discharges. Although the primary cause of TN must necessarily affect the primary afferents, the pathophysiological mechanism may or may not secondarily involve the central neurons.

Recent epidemiological studies of general practice research databases reported a TN incidence ranging from 12.6 to 26.8 per 100.000/year, with the incidence increasing with age (16.3 in the fourth decade, 30.6 in patients older than 80 years)[3, 4].

Pain distribution is unilateral (bilateral TN may sometimes occur in MS). The maxillary division is the most frequently affected, both in classic and symptomatic TN. Pain, usually referred to as stabbing or electric-shock-like, is brief and paroxysmal, lasting a few seconds. Paroxysms may be provoked by stimulating cutaneous or mucous trigeminal territories (trigger zones), regardless of the distribution of the perceived pain.

Carbamazepine, still remains the gold standard drug with the highest success rate. The second drug of choice recommended by international guidelines is oxcarbazepine. In a comparison between these two drugs, efficacy was shown to be very similar but tolerability is better with oxcarbazepine[1].
